# Long non-coding RNA BX357664 inhibits cell proliferation and metastasis in human lung cancer

**DOI:** 10.3892/ol.2021.12436

**Published:** 2021-01-05

**Authors:** Shu-Hui Xiao, Gong-Xiang Li, Lingli Quan

Oncol Lett 17: 2607-2614, 2019; DOI: 10.3892/ol.2019.9886

Following the publication of the above article, the authors have requested a change in the authorship on the paper, and the revised list of authors is presented above; essentially, the fourth author (Yunfeng Fu) has been removed from the paper on account of the article being submitted without his permission and knowledge. Yunfeng Fu was added as an author to thank him for the experimental and technical guidance provided in the implementation of previous projects, albeit without his authorization; furthermore, the authors now recognize that the inclusion of Dr Fu as an author did not meet with the criteria proposed by the International Committee of Medical Journal Editors (IJCME) for authorship on scientific articles. All the original authors (including Yunfeng Fu) agree with the revision made to the authorship on this paper, and the authors remaining on the paper apologize for any inconvenience caused. Therefore, the revised authors’ names and affiliations in this paper (including the revised Corresponding Author details) are as follows:

SHU-HUI XIAO^1^, GONG-XIANG LI^1^ and LINGLI QUAN^2^

^1^Department of Clinical Laboratory Medicine, The People's Hospital of Jinyi, Jinyi, Shanxi 710003; ^2^The First Department of Respiratory of Central Hospital of Zhuzhou, Zhuzhou, Hunan 412000, P.R. China

*Correspondence to:* Dr Lingli Quan, The First Department of Respiratory of Central Hospital of Zhuzhou, 116 South Changjiang Road, Zhuzhou, Hunan 412000, P.R. China

E-mail: jing1969wang@sina.com

